# Effect of Anthropogenic Disturbances on the Microbial Relationship during Bioremediation of Heavy Metal-Contaminated Sediment

**DOI:** 10.3390/microorganisms11051185

**Published:** 2023-04-30

**Authors:** Quanliu Yang, Shiqi Jie, Pan Lei, Min Gan, Peng He, Jianyu Zhu, Qingming Zhou

**Affiliations:** 1 College of Agronomy, Hunan Agricultural University, Changsha 410128, China; 2 School of Minerals Processing and Bioengineering, Key Laboratory of Biohydrometallurgy of Ministry of Education, Central South University, Changsha 410083, China

**Keywords:** heavy metal, sediment, anthropogenic disturbances, bioleaching, bioaugmentation

## Abstract

Soil, sediment, and waters contaminated with heavy metals pose a serious threat to ecosystem function and human health, and microorganisms are an effective way to address this problem. In this work, sediments containing heavy metals (Cu, Pb, Zn, Mn, Cd, As) were treated differently (sterilized and unsterilized) and bio-enhanced leaching experiments were carried out with the addition of exogenous iron-oxidizing bacteria *A. ferrooxidans* and sulfur-oxidizing bacteria *A. thiooxidans*. The leaching of As, Cd, Cu, and Zn was higher in the unsterilized sediment at the beginning 10 days, while heavy metals leached more optimally in the later sterilized sediment. The leaching of Cd from sterilized sediments was favored by *A. ferrooxidans* compared to *A. thiooxidans*. Meanwhile, the microbial community structure was analyzed using 16S rRNA gene sequencing, which revealed that 53.4% of the bacteria were *Proteobacteria*, 26.22% were *Bacteroidetes*, 5.04% were *Firmicutes*, 4.67% were *Chlamydomonas*, and 4.08% were *Acidobacteria*. DCA analysis indicated that microorganisms abundance (diversity and Chao values) increased with time. Furthermore, network analysis showed that complex networks of interactions existed in the sediments. After adapting to the acidic environmental conditions, the growth of some locally dominant bacteria increased the microbial interactions, allowing more bacteria to participate in the network, making their connections stronger. This evidence points to a disruption in the microbial community structure and its diversity following artificial disturbance, which then develops again over time. These results could contribute to the understanding of the evolution of microbial communities in the ecosystem during the remediation of anthropogenically disturbed heavy metals.

## 1. Introduction

Heavy metal pollution (HMP) is an important and urgent environmental problem, widespread in soil and aquatic systems, caused by natural and anthropogenic activities [1,2,3,4]. The nature of most HMP includes biotoxicity, persistence, and irreversibility. HMP can disrupt ecosystem function and cause the enrichment of heavy metal elements in human and biological tissues through the food chain, threatening human health [5,6]. In aquatic environments, heavy metal pollution is considered to be the most serious pollution [7,8]. Sediment plays a special role in transporting and storing noxious metals in aquatic environments [9,10]. Heavy metals are present in a variety of forms and matrices in sediment, resulting from the variation in pH, redox potential, nutrition, and other physicochemical characteristics in aquatic systems [11]. Microorganisms play an important role in the circulation of elements. Microorganisms can largely alter metal mobility, distribution, and toxicity. However, the toxicity is related to the valence status.

Plenty of treatment strategies have been adopted to remediate HMPs. They are designed for removing heavy metals from contaminated sites or reducing their bioavailability and mobility [12,13]. The primary strategies include physical, chemical, and biological technologies such as capping, adsorbents, washing, electrochemical remediation, phytoremediation, and microbial remediation [14,15,16,17,18,19]. However, most of the technologies proved to be inefficient, high-cost, and even risky for secondary contamination, which limited their further application [20,21]. Bioaugmentation is an alternative bioremediation strategy that works by adding exogenous microorganisms or by enhancing the relative abundance of particular indigenous microorganisms to degrade specific compounds. Since this approach is simple to perform, applicable in large areas, cost-effective, and highly efficient, bioaugmentation is recognized as an attractive approach to be widely used for HMP remediation [22]. In the past, there have been various studies on bioaugmentation systems. For example, bioleaching acidophilic microorganisms (*Acidithiobacillus ferrooxidans*, *Acidithiobacillus thiooxidans*) or neutrophilic microorganisms (*Aspergillus niger*) are the primary exogenous microorganisms to extract metals from tailings or heavy metal-contaminated soil [8,23]. An autochthonous soil consortium and an exogenous consortium were added to clayey or silty soil to bioremediate diesel fuel pollution. The results show that bioaugmentation with a combination of native and exogenous consortia can remove pollutants successfully [24]. Although there have been executed studies on bioaugmentation which were primarily focused on the remediation of contaminants, we still have only limited information about those interactions between native and exogenous microorganisms which determine the microbial evolution of adaptation. Our understanding and the adaptation of microorganisms to it are limited. Thus, a better understanding of the interactions between exogenic and endogenic microorganisms after anthropogenic disturbances is important to the ecosystem [25]. Environmental change can also adversely alter microbial functions and disturb the ecosystem profoundly. Therefore, it is necessary to monitor the development of biological communities to potential threats from anthropogenic disturbances, and to enhance the effectiveness of bioaugmentation.

Nowadays, next-generation sequencing of the small subunit of the ribosomal gene (SSU ribosomal DNA) has been applied frequently in surveys of microbial community structure and dynamic change. This technique offers an unprecedented opportunity to comprehensively examine the ecosystem’s response to environmental change [26,27]. Therefore, in this study, not only were the dynamics of pH/ORP and heavy metal leaching efficiency during bioremediation described, but also the evolution of microbial communities during the remediation of heavy metal-contaminated sediments under artificial disturbance. We used a random matrix theory (RMT)-based method to delineate phylogenetic molecular ecological networks of microbial communities based on high-throughput sequencing data, to analyze the impact of anthropogenic disturbances on community change during microbial remediation and its development, and to further improve the potential of microbial remediation of heavy metal-contaminated sediments. This is a powerful tool to elucidate network interactions in newly formed microbial communities. We focused on the following questions: (i) How does an anthropogenic environmental change such as exogenous microorganism addition affect the sediment microbial community diversity and structure? (ii) How does a newly formed microbial community adapt to environmental changes? (iii) What are the key populations in the newly formed environment? The results are supposed to give novel insight into the evolution of microbial communities in an anthropogenic-only disturbed ecosystem and to allow for the prediction of processes by which the microbial communities respond to environmental changes. These are fundamentally important for research in microbial ecology and environmental microbiology.

## 2. Materials and Methods

### 2.1. Sample Collection and Preparation

Heavy metal-contaminated sediments (~10 cm in depth) were collected from Xiangjiang River in Zhuzhou city, Hunan province, China (N 27.8554401, E 113.0786195). This is a typical industrial city. After collection, the samples were sent immediately to the laboratory. Any visible material (for example debris, animal, or plant residues) was removed manually from the sediment. Then, the samples were split into two subsets: one subsample was stored at −80 °C for microbial community analysis, and the other one was stored at 4 °C for geochemical soil variables measurements. That was followed by intensified bioleaching experiments. The pH and ORP of the sediment samples were determined with a PHS-3C pH meter (Leici, China) in a sediment-to-distilled water ratio of 1:2.5 (m/v). The concentrations of heavy metals in the sediment were determined by inductively coupled plasma-atomic emission spectrometry (ICP-AES).

### 2.2. Strains and Growth Conditions

The bacterial strains *Acidithiobacillus ferrooxidans* 23270 and *Acidithiobacillus thiooxidans DMC* were taken from the Key Laboratory of Biometallurgy of Ministry of Education, and used for bioaugmentation treatments. All strains were grown in 9K medium: ((NH_4_)_2_SO_4_ 3.0 g/L, KCl 0.1 g/L, K_2_HPO_4_·3H_2_O 0.5 g/L, MgSO_4_·7H_2_O 0.5 g/L, Ca(NO_3_)_2_ 0.01 g/L) with 4.47 g/L FeSO_4_·7H_2_O or elemental sulfur as supply energy, for *Acidithiobacillus ferrooxidans* and *Acidithiobacillus thiooxidans*, respectively. The strains were incubated at 30 °C and 170 rpm for 2 days. The concentration of each strain was 5.0 × 10^6^ cells/mL, as measured by microscopic counting.

### 2.3. Intensified Bioleaching Experiments

After air drying and sieving (200 mesh griddle), a part of the sediment samples was sterilized at 121 °C for 30 min. Bioleaching experiments were conducted in 250 mL shake flasks containing 100 mL 9K medium with 0.5 g sulfur and 0.5 g pyrite. H_2_SO_4_ and NaOH were used to adjust pH until it reached 7.0. The bioaugmentation leaching experiments consisted of four treatments, as shown in Table 1, where unsterilized sediment (5.0 g) and sterilized sediment (5.0 g) were added to different conical flasks and inoculated with two different microorganisms, *A. ferrooxidans* and *A. thiooxidans*, respectively. Each set of experiments was performed with 10 replicates. All assays were incubated at 30 °C for 21 days at 180 rpm. The pH, ORP, and metal dissolution rate of the bioleaching systems were monitored every day. Samples for microbial community structure analysis were collected on days 7, 14, and 21. Distilled water was added into the bioleaching system to compensate for water evaporation.

### 2.4. Preparation of Microbial Genomic DNA for Sequencing

Microbial genomic DNA was extracted from 0.5 g (wet weight) sediment for each sample at different stages of the bioaugmentation experiment using the FastDNA^®^ Spin Kit for Soil (116560-200; MP Biomedicals, USA) following the manufacturer’s recommendations. Total extracted DNA was assessed using a NanoDrop ND-1000 Spectrophotometer (NanoDrop Technologies Inc., Wilmington, DE, USA) to test the quality. The primers 515F (5′-GTGCCAGCMGCCGCGG-3′) and 806R (5′-GGACTACHVGGGTWTCTAAT-3′) were selected to amplify the V4 hypervariable regions of the 16S rRNA gene (Caporaso et al., 2012). The primers were tagged with Illumina MiSeq-specific adapters, barcode, pad, and linker sequences. All primers were synthesized by Eurofins/MWG (Huntsville, AL, USA). Overall, a 50 μL PCR-system was used, which consisted of 39 μL of ddH_2_O, 5 μL of 10 × PCR buffer, 1.5 μL of dNTP mixture 1.5 uL of each primer (10 μM), 0.5 uL of Taq DNA Polymerase High Fidelity (TaKaRa), and 1 μL of template DNA (20–30 ng/μL). All PCR reactions were prepared in a dedicated sterile hood. Thermal cycling conditions were as follows: 94 °C for 1 min followed by 30 cycles of 94 °C for 20 s, 57 °C for 25 s and 68 °C for 45 s, with a final extension step at 68 °C for 10 min. Next, 50 μL of the PCR product mixed with 10 uL Loading Buffer was used for agarose gel (2%) detection. Then, the target DNA bands were purified with an E.Z.N.A Gel Extraction Kit (D2500; OMEGA Bio-tek, Inc., Atlanta, GA, USA).

According to the MiSeq Reagent Kit Preparation Guide (Illumina, San Diego, CA, USA), libraries were diluted and pooled for cluster generation and sequence analysis on one lane of an Illumina MiSeq apparatus at the Key Laboratory of Biohydrometallurgy of Ministry of Education, where the library was sequenced using the manufacturer’s standard procedures. The purified mixture was diluted and denatured to obtain the 8 pM sample DNA library and mixed with an equal volume of 8 pM PhiX (Illumina). Finally, 600 μL of the mixture library was loaded with read 1, read 2, and the index sequencing primers [28] on a 300-cycle (2 × 150 paired ends) kit.

### 2.5. Data Analysis

According to its barcodes, readers were affiliated with their sample. Then the data were digested via de-multiplexing and quality filtering, denoise, chimera checking and removing, and data normalization. After that, UCLUST at a 97% similarity lever was used to cluster operational taxonomic units (OTU) [29]. Next, OTUs were assigned to their representative sequence by the Ribosomal Database Project (RDP) [30,31] and the OUT abundance table was constructed. The above processes were conducted in the Galaxy pipeline (https://usegalaxy.org/, accessed on 23 August 2022) [32,33].

Microbial community diversity was calculated using Simpson’s reciprocal index (1/D), Shannon Weaver index (H′), and Shannon evenness (J). Dissimilarity analysis of the microbial community distribution was performed based on Bray–Curtis dissimilarity and nonmetric multidimensional scaling (NMDS) to analyze similarities. All of the above were run in R (v.2.12.0; http://www.rproject.org/, accessed on 27 April 2023) or using an online pipeline (http://ieg.ou.edu/, accessed on 27 April 2023). The random matrix theory (RMT)-based methods to construct molecular ecological networks (MENs) were performed through the open access pipeline (http://ieg2.ou.edu/MENA, accessed on 27 April 2023).

## 3. Results

### 3.1. pH and Oxidation–Reduction Potential (ORP) Variation during Bioleaching

**Figure 1:**
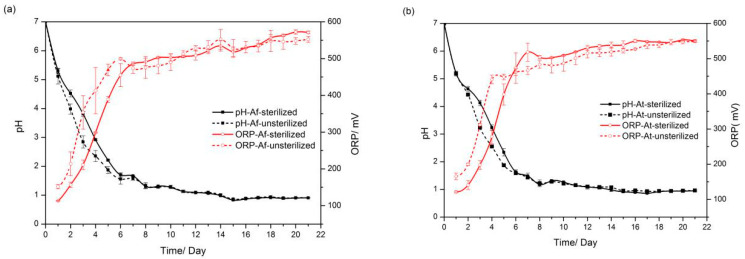
Variation in pH and ORP during bioleaching with the action of (**a**) *A. ferrooxidans* and (**b**) *A. thiooxidans*.

Environmental pH and redox potential exert an important influence on the migration of heavy metals, and therefore the dynamics of pH and ORP during bioleaching were examined. As shown in Figure 1, the pH decreased rapidly after the addition of exogenous microorganisms (*A. ferrooxidans* and *A. thiooxidans*) to the sediments (sterilized and unsterilized) for bioleaching. In the early stage, the pH of the group with unsterilized sediment added decreased relatively faster than that of the group with sterilized sediment added within 7 days. Afterward, the pH values of the four different treatment conditions gradually converged and decreased to approximately 0.9 within 21 days. The increase in ORP showed a similar trend to the decrease in pH. The ORP of the group with added unsterilized sediment increased more than the one with added sterilized sediment in the starting phase, and all were approximately equal in the later phase.

### 3.2. Solubilization of Heavy Metals

**Figure 2:**
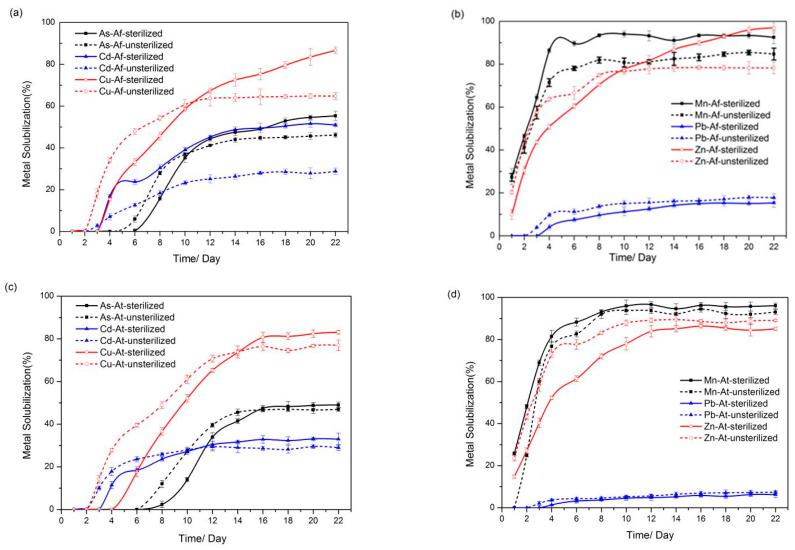
Solubilization of heavy metals during bioleaching: (**a**,**b**) bioleaching with *A. ferrooxidans*, (**c**,**d**) bioleaching with *A. thiooxidans*.

To investigate the concentration shifts of heavy metals (Cu, Pb, Zn, Mn, Cd, As) in sediments under microbial action, inductively coupled plasma atomic emission spectrometry (ICP-AES) was employed to determine the concentration of heavy metal ions in the leachate. As shown in Figure 2, the heavy metals in the sediment were dissolved to different degrees after 21 days of bioleaching, and the different treatment conditions led to discrepancies in the dissolution efficiency of heavy metals. On the whole, the leaching efficiencies of Cu, Mn, and Zn in the sediment were relatively higher than those of As, Cd, and Pb.

During the first 10 days of inoculation with *A. ferrooxidans*, As, Cu, and Zn were dissolved more rapidly in the unsterilized sediment than in the sterilized sediment, most likely owing to changes in environmental parameters such as pH and ORP caused by the combined action of *A. ferrooxidans* and the resident microorganisms in the sediment. However, the leaching rate of heavy metals was generally higher in sterilized sediments than in unsterilized sediments. The final dissolution rates of As, Cu, and Zn in the sterilized sediments reached 55%, 86%, and 96%, respectively, which were higher than the dissolution rates of As, Cu, and Zn in the unsterilized sediments (45%, 64%, and 78%, respectively). Additionally, the leaching of Cd and Mn in the sterilized sediment reached 50% and 92%, also higher than in the unsterilized sediment (29% and 84% solubility of Cd and Mn, respectively) (Figure 2a,b). A similar trend was observed in the system supplemented with *A. thiooxidans* (Figure 2c,d). The leaching rates of As, Cd, Cu, and Zn from the unsterilized sediments were higher in the early stages, while the dissolution rates of heavy metals from the sterilized sediments exceeded those from the unsterilized sediments in the final stages. Additionally, inoculation with *A. ferrooxidans* contributed significantly to the dissolution efficiency of Cd in sterilized sediments compared to the group inoculated with *A. thiooxidans*.

### 3.3. Microbial Community Structure

**Figure 3:**
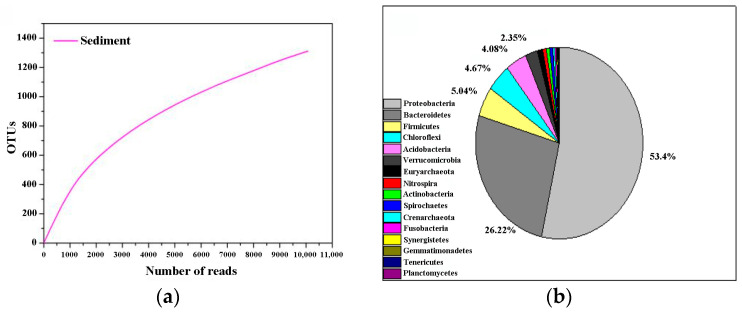
(**a**) Rarefaction curves of detected sequences in sediment sample; (**b**) the microbial composition at phylum level of the sediment sample.

**Figure 4:**
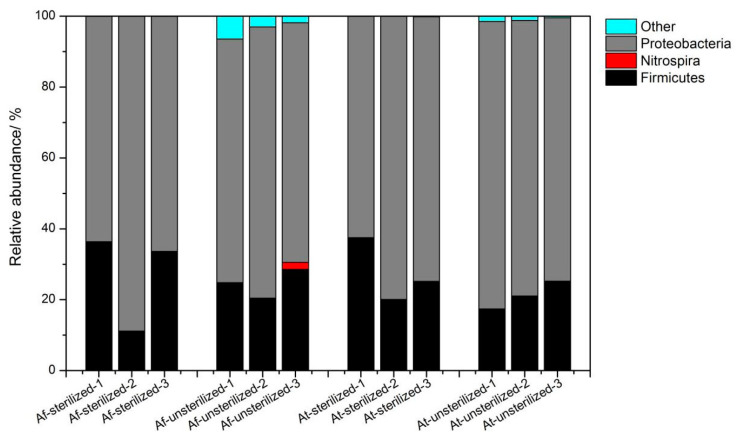
Relative abundance of phyla in bioleaching samples. “1” means in the sample from the 7th day, “2” means in the sample from the 14th day, and “3” means in the sample from the 21st day.

The bacterial community structure in the sediment of the bioaugmentation system (BS) was sampled once a week (three times). The samples were analyzed and compared with microbial communities in the original sediment (OSE). The taxonomic affiliation of these bacteria shows a high degree of biodiversity. A total of 2506 OTUs were detected in OSE and its rarefaction curves suggest that our sequencing efforts were sufficient for this study since the number of OTUs was almost saturated (Table 2, Figure 3a). Members of the phylum *Proteobacteria* were present predominantly in OSE, accounting for 53% of the total, followed by *Bacteroidetes* with a relative abundance of 26% and *Firmicutes* with 5% (Figure 3b). The comparison of the microbial taxonomic composition at the phylum level showed that *Proteobacteria* were dominant in both BS groups, whether sediment samples had been sterilized or remained unsterilized. The clone affiliation *Gammaproteobacteria* was the most represented class in our study. It ranged from 62% to 88%, which was attributed to the addition of a large number of exogenous *A. ferrooxidans* and *A. thiooxidans* (Figure 4). *Firmicutes* were the second dominant phylum in BS with a relative abundance which ranged from 11% up to 36%. Furthermore, compared to the OSE, it was shown that *Bacteroidetes* significantly decreased in BS to 0.003%~0.107%. *Bacteroidetes* and *Firmicutes* were most sensitive to adapting to the BS. Moreover, *Fusobacteria* (0.3%), *Synergistetes* (0.2%), *Gemmatimonadetes* (0.2%), and *tenericutes* (0.1%) were only retrieved from OSE, suggesting strong negative impacts of the multiple bioaugmentation treatments on these populations.

**Figure 5:**
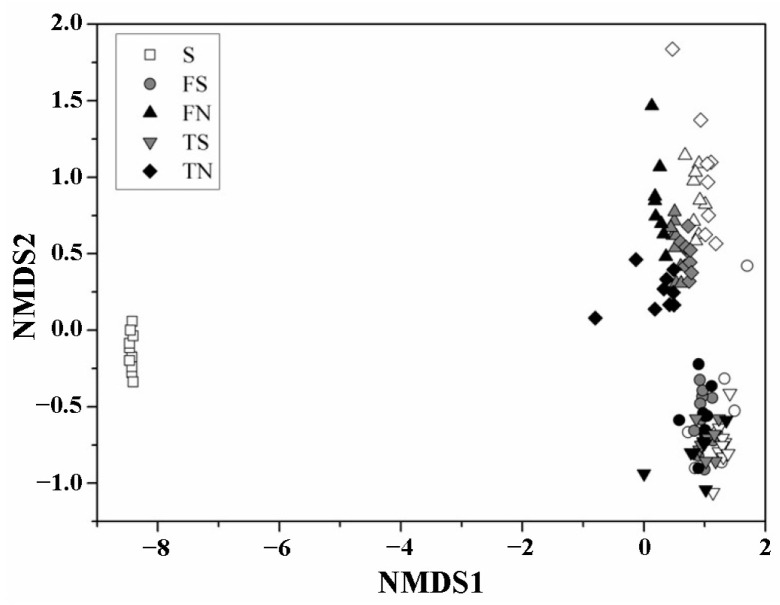
Nonmetric multidimensional scaling (NMDS) plots derived from pairwise unweighted UniFrac distances between the BS and OSE microbial community structures.

The overall pattern of microbial successions in the BS and OSE microbial communities are visualized on the first two coordinates of the nonmetric multidimensional scaling ordination based on the Bray–Curtis dissimilarity (Figure 5). As expected, the OSE clustered together, the sterilized sediment and the unsterilized sediment group from BS clustered separately and were distant from OSE. This finding is confirmed by the analysis of similarities, which show that the microbial community structures were significantly different for different sediment conditions.

### 3.4. Analysis of the Altered in Microbial Community with Temporal Turnover

During the 21-day operation of bioaugmentation, the bacterial community changed significantly. As revealed by alpha-diversities, which are shown by the Shannon–Weaver (diversity), evenness, and Chao indices, varied concurrently with species numbers. All microbial community indices indicated that diversity index showed a similar trend of fall–rise with bioaugmentation experiment operation (Table 3), and overall the groups containing unsterilized sediments (FN and TN) were richer in microbial diversity. The microbial time–decay relationships in taxonomic divisions were also estimated (Figure 5). As illustrated by the relative abundance of phyla for the bioaugmentation system, the *Proteobacteria* and *Firmicutes* in both sterilized sediment (FS, TS) and unsterilized sediment (FN, TN) assays showed a significant change with microbial temporal turnover. During the period between the 7th and 14th days, the relative abundance of *Proteobacteria* increased immediately in the FS, FN, and TS assays, but then decreased dramatically until the end, after 21 days of bioleaching. In contrast, the *Firmicutes* decreased during the period between the 7th and 14th days, and then increased until day 21 in FS, FN, and TS assays. The most obvious changes were noted for the sterilized groups (FS and TS), where the abundance of *Proteobacteria* changed from 63% (D7) to 88% (D14) and then to 66% (D21) in FS, from 63% (D7) to 80% (D14) and then to 75% (D21) in TS. The abundance of *Firmicutes* changed from 36% (D7) to 11% (D14) and then to 34% (D21) in FS or from 38% (D7) to 20% (D14) and then to 25% in TS. The TN showed a different trend during the period between the 7th and 14th days. The relative abundance of *Proteobacteria* continued to decrease from 81% to 74% and the *Firmicutes* increased from 17% to 25%. The above phenomena may have been caused by different treatments, which led to different proportions of microorganisms in the systems.

**Figure 6:**
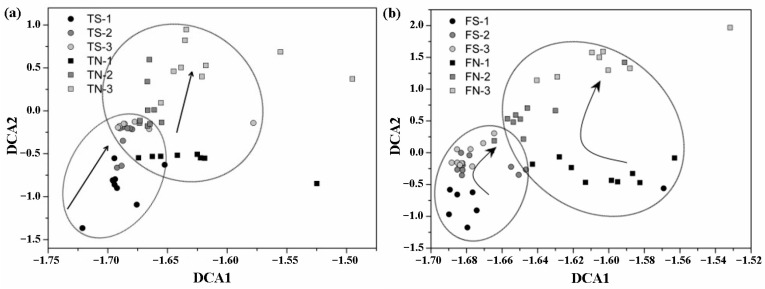
DCA analysis of microbial communities of bioleaching samples: (**a**) bioleaching with *A. thiooxidans*; (**b**) bioleaching with *A. ferrooxidans*. “1” means the sample of 7th day, “2” means the sample of 14th day, “3” means the sample of 21st day.

To explore the overall variability in the community composition at the different stages, detrended correspondence analysis (DCA) was performed to reveal the community structure. As shown in Figure 6, the parallel samples belonging to the same group cluster together, indicating that the experiment is repeatable. Additionally, the microbial communities in the same group of samples had different compositional changes at different stages. In addition, the microbial community structure under disinfected sediment and un-disinfected sediment treatment conditions was also significantly different and dynamic with time.

### 3.5. Species with Significant Variation

**Figure 7:**
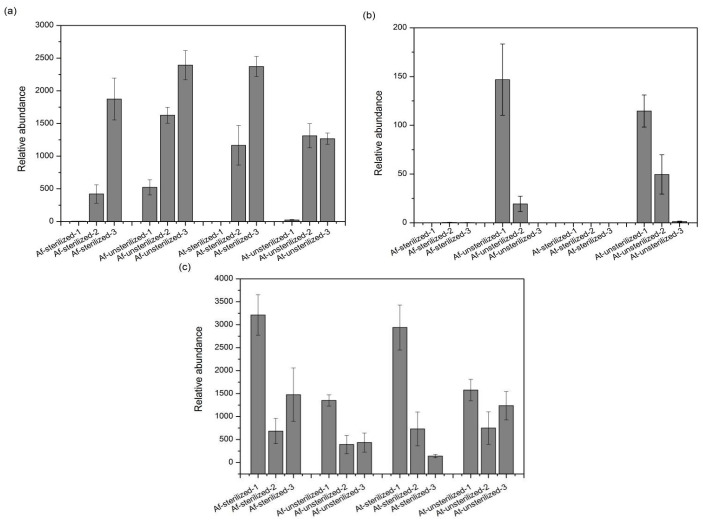
Relative abundance of the dominant genera in bioleaching samples: (**a**) *Sulfobacillus*; (**b**) *Acidiphilium*; (**c**) *Alicyclobacillus*. “1” means the sample from the 7th day, “2” means the sample from the 14th day, “3” means the sample from the 21st day.

The changes in species composition and diversity during the experiment should be a response to the environmental changes caused by the addition of exogenous microorganisms (*A. ferrooxidans* and *A. thiooxidans*). Among the bioaugmentation treatment of the heavy metal-contaminated sediment from Xiangjiang River, the presence of several microorganism contents has changed significantly (Figure 7). From calculation at the taxonomic genus level, the content abundance revealed a remarkable increase for *Sulfobacillus* in TN, which increased from day 7 to day 14 and remained approximately at that level until day 21. Interestingly, through 16s rRNA gene sequence analysis, only a few *Sulfobacillus* were detected in OSE and BS at day 7. We suspect that the bioaugmentation contributed to the recovery of Bacillus or facilitated the *Sulfobacillus* bacterial capability of adapting to a new environment-induced explosion. The *Acidiphilium* genus decreased in the unsterilized assays and were almost not detectable anymore in sterilized groups, suggesting that our ways of bioaugmentation changed the conditions causing *Acidiphilium* to become outcompeted. The genus *Alicyclobacillus* showed a similar trend and disappeared, except for in TS groups.

**Figure 8:**
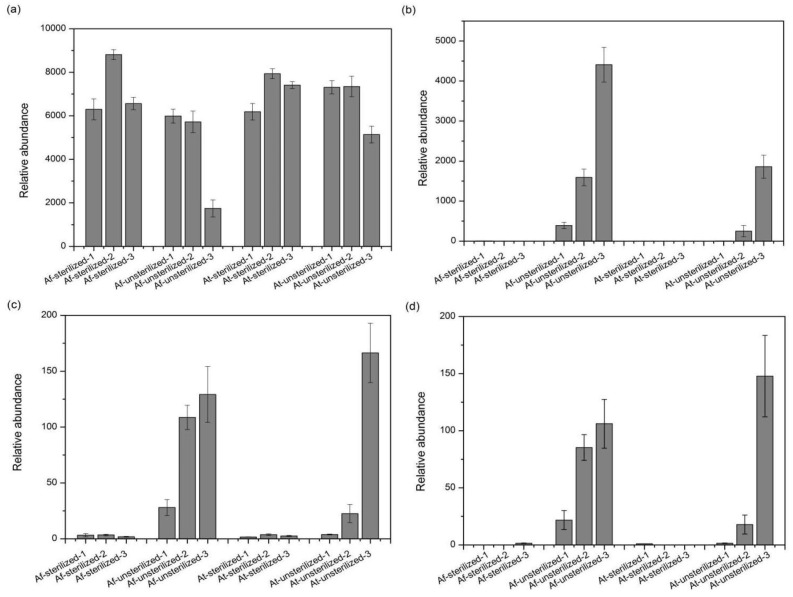
Relative abundance of some OTUs belonging to *Acidithiobacillus* in bioleaching samples: (**a**) OTU_1; (**b**) OTU_3; (**c**) OTU_2414; (**d**) OTU_2723. “1” means the sample from the 7th day, “2” means the sample from the 14th day, and “3” means the sample from the 21st day.

OTUs 1, 3, 2414, and 2713 affiliated with the *Acidithiobacillus* genus show that the variation trend of OTU 1 is consistent with the previous *Proteobacteria* altered trend; thus, we deduced that the OTU 1 is likely to be affiliated with exogenous bacteria. The data in Figure 8 indicate that OTUs 3, 2414, and 2723 of the sterilized assays never grow; however, these OTUs grew well in the unsterilized test, and therefore they should be considered as native microbial species in the sediment.

### 3.6. Network Analysis

**Figure 9:**
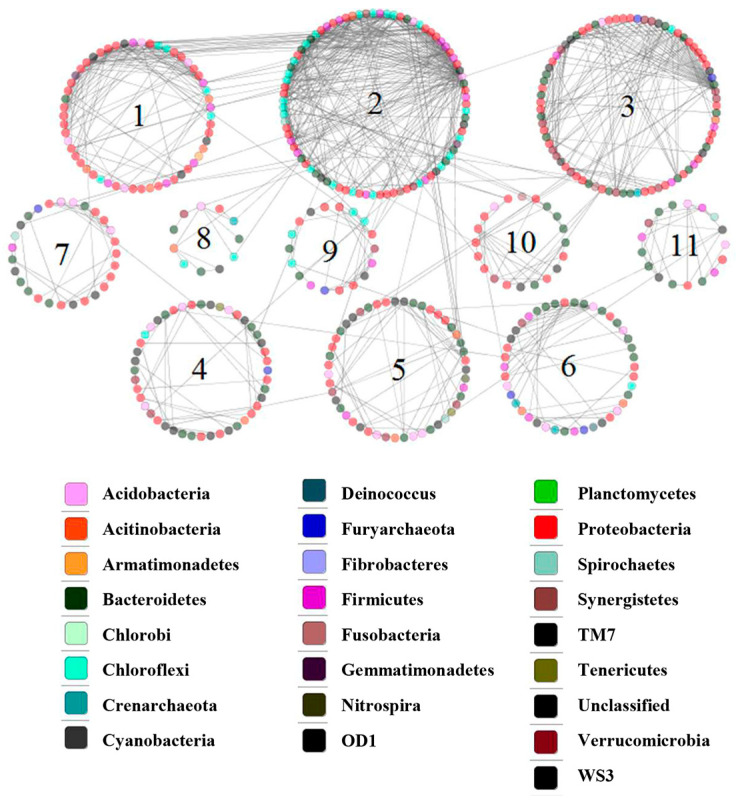
The pMENs of OTUs in the samples from Xiangjiang River sediment with 16S rRNA gene-based metagenomic data.

**Figure 10:**
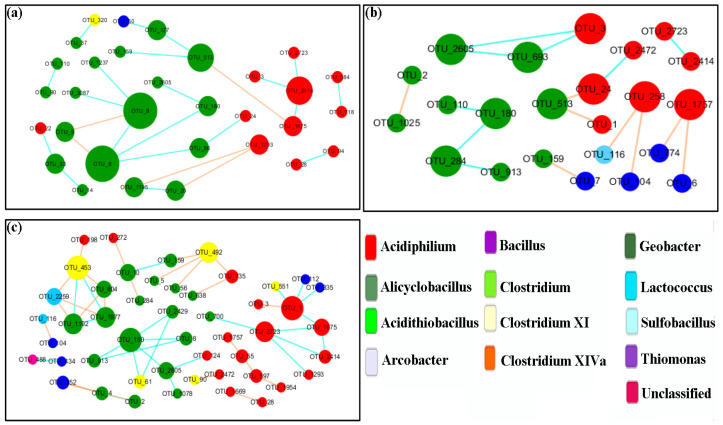
Network interactions between *Sulfobacillus*, *Acidiphilium, Alicyclobacillus*, and *Acidithiobacillus* in unsterilized sediment assay with the addition of *A. ferrooxidan*. (**a**) The sample from the 7th day; (**b**) the sample from the 14th day; (**c**) the sample from the 21st day.

**Figure 11:**
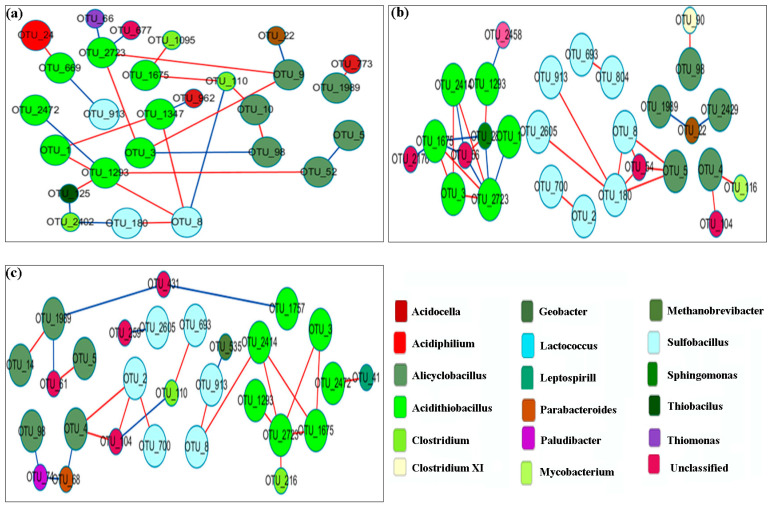
Network interactions between *Sulfobacillus*, *Acidiphilium, Alicyclobacillus*, and *Acidithiobacillus* in unsterilized sediment assay with the addition of *A. Thiooxidans*. (**a**) The sample from the 7th day; (**b**) the sample from the 14th day; (**c**) the sample from the 21st day.

We analyzed the phylogenetic diversity data for various sediment conditions (OSE and BS), using the RMT-based molecular ecological network method to illustrate the ecological interaction levels in terms of overall network topology, network composition, node overlap, and module preservation. As illustrated in Table 4, 2506 OTUs were detected plus 523 nodes with an average clustering coefficient (avgCC) of 0.157, and an average path distance (GD) of 3.406 in OSE. The data suggest a complicated relationship between the strains. Moreover, 11 submodules were determined (Figure 9), suggesting that the OSE comprised a high bearing capability of the microbial ecosystem. Table 4 shows the number of OTUs remaining in FN and TN at various stages, respectively. After scanning the thresholds through an RMT-based approach, the phylogenetic networks under sterilized assays were constructed with close similarity thresholds (*s_t_*) for FN (0.81) and TN (0.78). As illustrated in Table 4, the nodes and links at first decreased and later on increased. This suggests that the interaction between microbes became weakened in the first stage and was restored in the late stage. In Figure 10 and Figure 11, the majority of the nodes in the six networks belonged to *Proteobacteria* and *Firmicutes.* Obviously, these groups play a key role in networks. Considering the relative proportions of the four genera *Sulfobacillus*, *Acidiphilium*, *Alicyclobacillus*, and *Acidithiobacillus*, it can be deduced that they show a significant change over time. Taking into account the FN groups, *Acidiphilium* exhibited fewer interactions with others in the networks and vanished after 21 days of processing, while the *Acidithiobacillus* and the *Sulfobacillus* played an active role in the networks. Moreover, the abundance of *Alicyclobacillus* showed a “down and up” tendency (Figure 10). The majority genera *Sulfobacillus*, *Alicyclobacillus*, and *Acidithiobacillus* established a functioning interaction network with each other in the late stage, as depicted by a complex interaction after 21 days of bioleaching, suggesting that the changed environment resulted from bioleaching, which facilitated interactions between indigenous and exogenous microorganisms. As demonstrated in Figure 11, which is takes into account the TN groups, *Alicyclobacillus* and *Acidithiobacillus* had more complex interactions with each other on day 7, but almost vanished on day 14, while the interactions reappeared on day 21 of bioleaching. Moreover, *Acidiphilium* appeared only on day 7 and disappeared during bioleaching. These observations coincide with the aforementioned results (Figure 11). According to the results above, it can be conducted that a certain interaction network had formed between the exogenous and native microorganisms.

## 4. Discussion

Two types of exogenous microorganisms were added to sediments as controls; the sediments were inactivated without the addition of *A. ferrooxidans/A. Thiooxidans* (FS, FN, TS, and TN). The results indicated that the pH decreased during the first 6 days. Moreover, the unsterilized sediment assays (FN and TN) did not decrease (FS and TS). However, the pH in all assays was finally reduced to the same value in the later stage. The increase in the ORP showed a similar trend with pH. These results support that the synergistic effect between indigenous microorganisms and exogenous microorganisms under anthropogenic disturbance has been produced in the initial stage, but the ecosystem restoration ability plays a leading role in the later stage of recuperation from environmental disruption.

The data of ICP-AES analysis indicated that the sediment bacterial community responded differently to contamination with heavy metals and multiple bioaugmentation treatments. The dissolution rates of different metals have various leaching efficiencies in the four groups, and it is indicated that the different synergistic effects of microorganisms in substrate utilization can bring different impacts on metal solubilization. Meanwhile, the bioleaching effect of various heavy metals in this work was stable and the sediment treatment was much larger than the previously reported enhanced system with the exogenous addition of sulfur-oxidizing microflora [34].

Unlike previous biofortification systems [35] that focused too much on the leaching effect of heavy metals, the study of microbial communities in biofortified systems after anthropogenic disturbance is also a focus of attention. Due to its value in bioremediation of contaminated and microbial ecology, the microbial community composition and structure as well as their ecological function are sensitive in response to environmental changes under continuous bioleaching with bioaugmentation, and are of great significance in scientific research. The sediment microbial community can be shaped by environmental factors. A remarkable differentiation of the bacterial community structure OSE and BS was detected based on NMNS. The data indicate that the inoculation with *A. ferrooxidans* or *A. Thiooxidans* bioaugmentation changed the original microbial community structure. The close clustering of samples in OSE or other groups indicates that the samples from original sediments and bioaugmentation treatments contained a similar microbial composition in one system. The different treatments of the sediment (sterilized or unsterilized) are the major determinants for the discrepancy in community composition.

The results of the OTU-based analysis show that the richness of microorganisms has a similar trend during bioleaching. Shannon diversity indices, evenness, and Chao values were also used as indicators. In regard to the microbial taxonomic composition and content variations for bioleaching, the relative abundance of *Proteobacteria* first decreased, then increased, but for *Firmicutes* the opposite was noted. Consequently, one perspective assumes that the relative abundance of *Proteobacteria* in TN reached its highest value before the seventh day. In addition, the genus *Sulfobacillus* disappeared after sample sterilization, and reappeared later on. According to the literature, *Sulfobacillus* needs elevated levels of CO_2_, yeast extract, or a close association with heterotrophic iron-oxidizing bacteria such as *Acidimicrobium ferrooxidans* for good growth [36,37]. Thus, we consider the increase in *Sulfobacillus* at later stages to be associated with the addition of exogenous bacteria. The relative abundance of *Acidiphilium* declined in FN and TN, which perhaps can be attributed to competitive inhibition by the addition of sulfur oxidizers such as *A. ferrooxidans* or *A. Thiooxidans*. In summary, the results indicate the differences in sensitivity of the microbial taxonomic groups to the disturbances in the environment. The four different OTUs belonging to *Acidithiobacillus* showed a larger variation during bioleaching. According to the trends of OTUs 3, 2414, and 2723, the environmental carrying capacity of the sterilization group was weakened, which is most probably related to its artificially reduced species diversity. In addition, there are potential synergistic effects between native and exotic microorganisms to help them to jointly cope with stresses such as heavy metal stress and changes in the survival environment due to anthropogenic disturbances.

The relationship between native and exotic microbes is a critical issue in ecosystems. However, due to the extreme complexity of such ecosystems, it is a difficult aspect to understand. As illustrated in this study, the relationship between microbial interactions and the key population can be identified based on network topology, node overlap, and module preservation. According to the results of the molecular network analyses, we detected that the original sediments contain a complex interaction network. The bioleaching samples are supported by nodes, links, and GD values, as the criterions show a simple network construct as compared to the original sediment, and show a “down and up” trend. According to the results, it was found that a certain degree of weakening of biodiversity-affecting ecosystem functions occurs under human disturbance. In the early stages of bioleaching, microbial interactions were weakened. After adaptation to the acidic environment, a portion of the indigenous microorganisms adapted and integrated into the interaction network.

## 5. Conclusions

In this study, *A. ferrooxidans* and *A. thiooxidans* were added to the sediment, and different treatments (high-pressure steam sterilization and without sterilization) were conducted for bio-enhanced leaching experiments. In the early stage during bioleaching, the pH decline rate for the unsterilized assay was significantly greater than that for the sterilized assay. In the bacterial group, the difference between the pH values in the two groups was reduced, and the pH in the post-leaching sterilization group was smaller than that in the unsterilized group. ORP shows the same trend as pH. It indicates that the microbial communities of different groups will reach a certain steady state after a certain time of artificial disturbance. In addition, in the bioaugmented bioleaching system, the dissolution rates of Cu, As, and Zn were higher in the unsterilized group at the beginning of the experiment, indicating that the interaction between exogenous and native microorganisms can promote the leaching of heavy metals to some extent. The 16S rRNA gene sequencing technology was used to analyze the microbial community structure of sediment and leached samples. Overall, 53.4% of the bacteria in the sediment belonged to *Proteobacteria*, 26.22% belonged to Bacteroidetes, 5.04% were *Firmicutes*, 4.67% were *Chlamydomonas*, 4.08% were *Acidobacteria*, and 2.35% were *Verrucomicrobia.* The DCA analysis showed samples that were clustered together, and the abundance (diversity and Chao value) of microorganisms in leaching samples showed dynamic changes over time. The increase in the relative abundance of *Sulfobacillus* and the rapid decrease in the relative abundance of *Acidiphilium* in the unsterilized group also indicated that the microbial community structure was constantly adjusting to the survival environment after artificial disturbance. Network analysis showed that there was a complex network of interactions in the diapause, and the interactions between microscopic organisms were weakened in the early stages of leaching, while the growth of some locally dominant bacteria increased the interactions between microorganisms after adaptation to acidic environmental conditions, allowing more bacteria to participate in the network and form tighter relationships. The relationship between native and exotic microorganisms and the change in microbial communities under artificial disturbance are key issues in the ecosystem. This study shows that the microbial community structure and its diversity, which were destroyed by artificial disturbance, will recover with time, and the linkage between exotic and native microorganisms will gradually be established and developed more and more closely to promote the removal of heavy metals from the sediment. These findings contribute to the understanding of the evolution of microbial communities in ecosystems during the remediation of anthropogenically disturbed heavy metals. However, the mechanisms of microbial remediation effects on heavy metals in sediments under anthropogenic disturbance and the synergistic effects within microbial communities still need further research in the future; this will aid in enhancing the potential of the microbial remediation of heavy metal-contaminated sediments, and in providing new breakthroughs for advancing sustainable solutions for the remediation of heavy metal-contaminated sediments.

## Figures and Tables

**Table 1:** Description of the bioleaching experiments.

Groups	Sediment Type	Inoculated Bacteria
FS	sterilized sediment	*Acidithiobacillus ferrooxidans*
FN	unsterilized sediment	*Acidithiobacillus ferrooxidans*
TS	sterilized sediment	*Acidithiobacillus thiooxidans*
TN	unsterilized sediment	*Acidithiobacillus thiooxidans*

**Table 2:** The diversity, evenness, Chao indices, and number of OTUs in sediment samples.

Item	Diversity	Evenness	Chao	Number of OTUs
Value	5.73008	0.80073	2071.197	2506

**Table 3:** The diversity, evenness, Chao indices, and number of OTUs in bioleaching samples.

Index	*A. ferrooxidans* + Sterilized Sediment			*A. ferrooxidans* + Unsterilized Sediment		
	1	2	3	1	2	3
Diversity	1.00745	0.44935	0.88317	1.54914	1.32424	1.50363
Evenness	0.31305	0.28736	0.28511	0.36462	0.32895	0.36427
Chao	40.00521	39.10833	57.80833	120.71262	110.67991	137.32339
Number of OTUs	81	34	80	176	116	137
**Index**	***A. thiooxidans* + sterilized sediment**			***A. thiooxidans* + unsterilized sediment**		
	**1**	**2**	**3**	**1**	**2**	**3**
Diversity	0.95649	0.62382	0.67928	1.19243	0.99909	1.40654
Evenness	0.34091	0.21024	0.21861	0.27989	0.24985	0.33742
Chao	33.50001	26.65953	88.11191	215.86589	88.60981	219.89771
Number of OTUs	53	54	116	230	125	247

“1” means the 7th day, “2” means the 14th day, “3” means the 21st day.

**Table 4:** Topological properties of the pMENs of microbial communities in sediment.

Index	Number of Original OTUs	Similarity Threshold	Total Nodes	Total Links	R Square of Power-Law	Average Clustering Coefficient (avgCC)	Average Path Distance (GD)	Module	Modularity
Sediment	2506	0.88	345	523	0.921	0.157	3.406	43	0.736
FN-1	176	0.81	27	22	0.74	0.16	0.316	7	0.764
FN-2	116	0.81	23	16	0.99	0.13	0.134	8	0.851
FN-3	136	0.81	45	43	0.97	0.219	0.226	10	0.829
TN-1	230	0.78	41	49	0.85	0.128	3.447	6	0.62
TN-2	125	0.78	33	33	0.85	0.184	0.265	9	0.707
TN-3	247	0.78	39	36	0.9	0.082	0.638	8	0.771

“1” means the sample from the 7th day, “2” means the sample from the 14th day, and “3” means the sample from the 21st day.

## Data Availability

All of the relevant data are provided in the form of regular figures and tables.
